# Predators Reduce Extinction Risk in Noisy Metapopulations

**DOI:** 10.1371/journal.pone.0011635

**Published:** 2010-07-21

**Authors:** James C. Bull, Michael B. Bonsall

**Affiliations:** 1 Populations and Disease Group, Department of Biological Sciences, University of Warwick, Coventry, United Kingdom; 2 Mathematical Ecology Research Group, Department of Zoology, University of Oxford, South Parks Road, Oxford, United Kingdom; Centre National de la Recherche Scientifique, France

## Abstract

**Background:**

Spatial structure across fragmented landscapes can enhance regional population persistence by promoting local “rescue effects.” In small, vulnerable populations, where chance or random events between individuals may have disproportionately large effects on species interactions, such local processes are particularly important. However, existing theory often only describes the dynamics of metapopulations at regional scales, neglecting the role of multispecies population dynamics within habitat patches.

**Findings:**

By coupling analysis across spatial scales we quantified the interaction between local scale population regulation, regional dispersal and noise processes in the dynamics of experimental host-parasitoid metapopulations. We find that increasing community complexity increases negative correlation between local population dynamics. A potential mechanism underpinning this finding was explored using a simple population dynamic model.

**Conclusions:**

Our results suggest a paradox: parasitism, whilst clearly damaging to hosts at the individual level, reduces extinction risk at the population level.

## Introduction

Subdividing habitats results in spatially structured metapopulations and affects the dynamics of species that occupy them [1]–[5]. The early ‘blinking lights’ model [2] of metapopulation dynamics makes a number of simplifying assumptions, notably that habitats effectively comprise an infinite numbers of patches, with equal probability of dispersal between any pair of patches, and that within patch (local) population dynamics are fast (instantaneous) compared to metapopulation dynamics, so that local populations may be considered to be in one of two equilibrium states, either at the (static) local carrying capacity of the habitat patch or locally extinct. These assumptions make the classic model of metapopulations particularly unsuitable for describing the population dynamics of small metapopulations, with few patches and/or low abundances where stochastic processes may be more influential.

A number of theoretical [6]–[8] and empirical [9], [10] studies have investigated patterns of metapopulation synchrony in larger habitats with local movement and, importantly, where abundance fluctuations are under deterministic control (limit cycles, chaos) but this unique investigation explores the dynamics of noise-driven, small metacommunities. Noise and stochasticity has an important role in many environmental and ecological processes. Events such as the invasion and expansion of species into new ranges, emerging diseases and extinction of vulnerable species of conservation or economic importance are likely to take place in restricted, spatially heterogeneous environments, where chance encounters between few individuals at random times will have a substantial impact on subsequent population dynamics.

**Figure 1:**
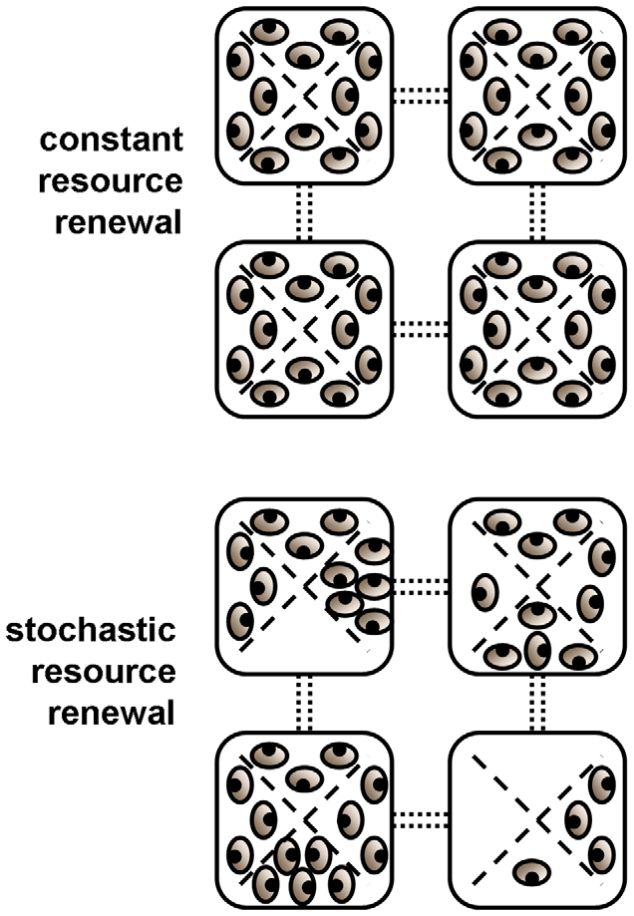
Plan view of four patch, square lattice, metapopulation design: dotted connectors indicate dispersal routes between patches (boxes); dashed lines represent barriers separating weekly host resource (bean) quantities but which did not impede insects. Beans were renewed on a four week regime with either constant or stochastic renewal. Long term average resource levels were two, three or four beans per patch, depending on renewal treatment (average three beans shown).

Here, using a well-characterised empirical system [11]–[14], we explored the replicated metapopulation dynamics of two and three species host-parasitoid assemblages, using the bruchid beetles, *Callosobruchus maculatus*, *C. chinensis*, and the parasitoid, *Anisopteromalus calandrae*. Simple experimental designs of linked patches, connected for limited periods of time, allowed metapopulations to be constructed with two species, host-parasitoid and three species, apparent competitive [15], [16] community modules over a range of environmental conditions (Figure 1). We aimed to understand the metapopulation dynamics of these resource-consumer interactions by simultaneously partitioning the contributions of local regulatory processes (patch processes), space (regional dynamics) and stochasticity. Contrasting these specific community modules [17] allows the unique opportunity to quantify the effects of community complexity (two *vs*. three species) without introducing any additional types of species interactions (e.g. interspecific competition).

By capturing the metapopulation dynamics operating in these systems we were able to test, and support, the hypothesis that assemblage complexity is positively associated with spatial asynchrony. We explored a mechanistic basis by which predation can drive asynchronous dynamics and reduce metapopulation extinction risk using a spatially explicit population dynamic model. Our findings show that even when deterministic dynamics predict stable equilibria, highly mobile foragers can act on stochastic prey abundance fluctuations to reduce regional extinction risk.

## Methods

### Experimental setup

Replicated (*n* = 4) laboratory microcosms were used to explore the metapopulation dynamics of two and three species, host-parasitoid assemblages, using the bruchid beetles, *Callosobruchus maculatus*, *C. chinensis*, and the parasitoid, *Anisopteromalus calandrae*. Full details of the experimental setup have been published elsewhere [11]–[14]. Briefly, clear, plastic boxes (73×73×30 mm) were used as the baseline ‘patch’ for the study. Patches were connected for 2hrs each week to allow limited dispersal. All insect species present were readily able to pass through tubes connecting these habitat patches. In apparent competition modules, host species were separated in ‘double-decker’ stacked lattices, where a mesh floor/ceiling barrier prevented direct competition between host species but did not impede parasitoid movement between host species.

The host resources used in our study were black-eyed beans (*Vigna unguiculata*). A total of four different bean renewal regimes were implemented in a factorial design: treatment ‘a’ was a weekly regime of three beans per patch; treatment ‘b’ incorporated a long term average of three beans per patch but delivered stochastically (independent Poisson distributed) each week; treatment ‘c’ involved *C. maculatus* receiving two beans per patch and *C. chinensis* receiving four beans per patch (thus favouring the inferior host competitor); and in treatment ‘d’ the average resource levels of treatment ‘c’ were delivered stochastically (Poisson) over successive weeks.

Following an initial period for populations to establish, time series for all species were obtained by counting both alive and dead insects each week from every patch (dead insects were then removed). All experiments were undertaken in controlled environmental conditions (30°C, 70% relative humidity, 16 ∶ 8 light ∶ dark cycle).

### Statistical analysis

Our aim was to decompose multispecies metapopulation dynamics into local regulatory processes, the contribution of (host and parasitoid) dispersal and stochasticity. *Callosobruchus maculatus* was the superior competitor in the apparent competition interaction and survived to the end of the experimental period in the majority of replicates. Therefore, we are best able to explore the processes of population limitation in the presence of parasitoids with these *C. maculatus* metapopulations. Time series of adult abundances were used to parameterise a population dynamic model. This statistical model took the form of a time-delayed linearization of the Ricker model [18],
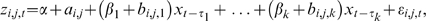
(1)where net reproductive rate, 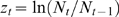, and *x_t−τ_* = 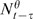, adult abundance lagged by τ weeks. *θ* is the optimal Box-Cox (power) transformation for a given lagged time series [19]. We considered time lags of up to four weeks (this being slightly longer than a single host generation). In order to improve temporal resolution, we calculated half-week lags through linear interpolation [20]. *α* represents the average value of the intercept and *β_k_* the average values of the *k* individual slopes for each time lagged adult abundance covariate.

This deterministic core was embedded within a hierarchical statistical framework: *i* patches (*i* = 1,…,4), nested within *j* metapopulation replicates (*j* = 1,…,4). The parameters *a_i,j_* and *b_i,j,k_* represent deviations between patches and metapopulations in each of *α* and *β_k_* respectively. *ε_i,j,t_* represents normally distributed residual errors, including the appropriate variance-covariance matrix to describe spatial correlation at the patch level.

The correct time lag structure and spatial correlation was identified by calculating Akaike Information Criterion (AIC) weights (*w_m_* = 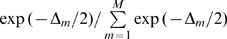 for *M* competing models. Evidence ratios (*w_m_*/*w_m′_*) greater than 2.718 indicated a substantial improvement in fit of model *m* over model *m′*, being equivalent to a difference in AIC (Δ*_m_*) of greater than two [21]. All statistical analyses were performed using the R statistical package (http://www.r-project.org) for which the code is available on request.

### Mechanistic population modelling

Simulated host-parasitoid dynamics were modelled as a discrete time Nicholson-Bailey coupled system. The single, non-trivial (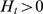) equilibrium is unstable in this model. However, the trophic interaction can be stabilised by allowing a fixed proportion, *S*, of hosts to escape parasitism and survive to the next time step [22]. This stabilising mechanism is independent of host self regulatory (density-dependent) processes. In contrast to previous studies [6]–[10], our primary aim was to investigate spatial correlation between stochastic fluctuations, rather than deterministic, nonlinear dynamics (limit cycles, chaos). Therefore, we allowed a sufficient fraction of hosts to survive between time steps in order to ensure damped oscillations in host dynamics.

We embedded these local population dynamic processes within a two patch metapopulation, representing the simplest spatially explicit habitat. Environmental stochasticity was modelled by adding spatially uncorrelated, density independent noise on host population growth rate at each time step. Our coupled host-parasitoid dynamics were described by:
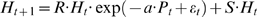
(2)

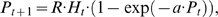
(3)where *R* is host fecundity, *a* is the parasitoid attack rate and noise, 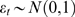.

Here, *R* = 2.718, *a* = 0.1, *S* = 0.7 and patches were initially populated with 100 hosts and 100 parasitoids. Sensitivity analysis indicated that our results were qualitatively dependent on the deterministic population dynamics, i.e. parameter combinations which produced a stable equilibrium rather than cyclic dynamics, as opposed to specific parameter quantities.

The effects of host and parasitoid dispersal were investigated by allowing fixed (density independent) proportions of species to move between two identical (in terms of regulatory dynamics) local habitat patches at each time step. Host and parasitoid dispersal rates were varied independently. Simulations were run for 1000 time steps, with the first 500 points discarded to allow equilibrium dynamics to establish, and replicated 100 times for each host-parasitoid dispersal proportion combination.

## Results

### Extinction risk

**Figure 2:**
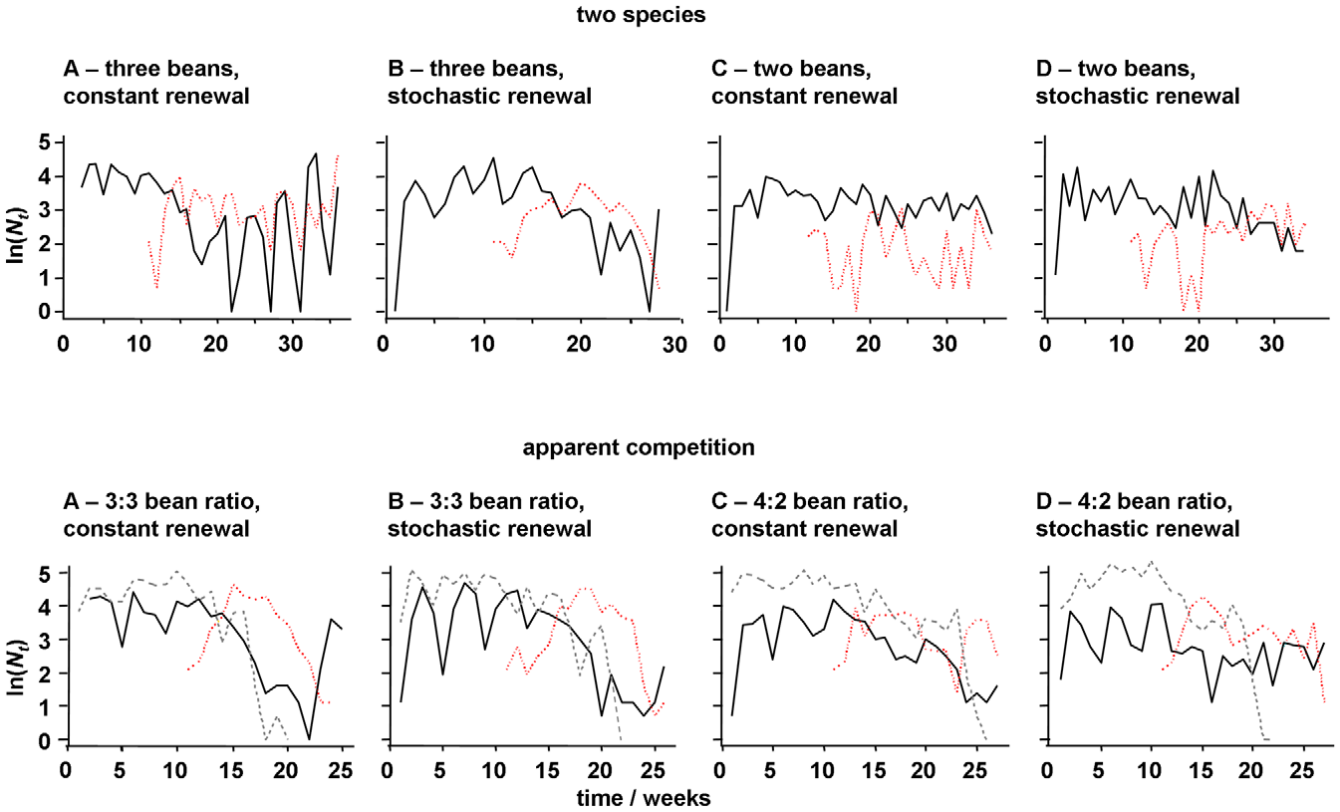
Representative time series of total adult abundances, from the replicated resource-consumer metapopulations, under different resource renewal regimes. Top row: host (*C. maculatus*, solid line) – parasitoid (*A. calandrae*, dotted line) interaction (patchwise weekly host resource renewal: (a) constant three beans, (b) Poisson average of three beans, (c) constant two beans and (d) Poisson average of two beans). Bottom row: two host species (*C. maculatus*, solid line; *C. chinensis*, dashed line) and one parasitoid species (*A. calandrae*, dotted line) apparent competition interaction (patchwise weekly host resource renewal: (a) constant 3∶3 ratio of beans per host species, (b) Poisson average 3∶3 ratio of beans per host species, (c) constant 4∶2 ratio, *C. chinensis* ∶ *C. maculatus*, of beans and (d) Poisson average 4∶2 ratio, *C. chinensis* ∶ *C. maculatus*, of beans).

Representative time series from two species, host-parasitoid and three species, apparent competition interactions are shown in Figure 2. We modelled fluctuations in local host and parasitoid abundances using coefficients of variation as a response variable, scaling absolute fluctuations by population size. This provides a measure of stochastic extinction risk in a range of persistent populations. Environmental stochasticity increased levels of temporal variation in host abundance (log odds ratio (SE) = 0.0348 (0.0117), *t*_108_ = 2.96, *p* = 0.0038), although not parasitoid abundance (Likelihood ratio (L. R.) = 1.12, *p* = 0.29). Conversely, higher average host resource level did not influence levels of variation in the host population (L.R. = 0.00764, *p* = 0.93) but did increase parasitoid fluctuations (log odds ratio (SE) = 0.224 (0.0663), *t*_110_ = 2.48, *p* = 0.015). Most importantly, presence of an apparent competitor was not associated with changes in the levels of variation in host or parasitoid abundance (host: L.R. = 0.00764, *p* = 0.93; parasitoid: L.R. = 0.0943, *p* = 0.76): increased parasitism did not result in a greater chance of local host population extinction through stochastic fluctuations.

### Local population dynamics

**Figure 3:**
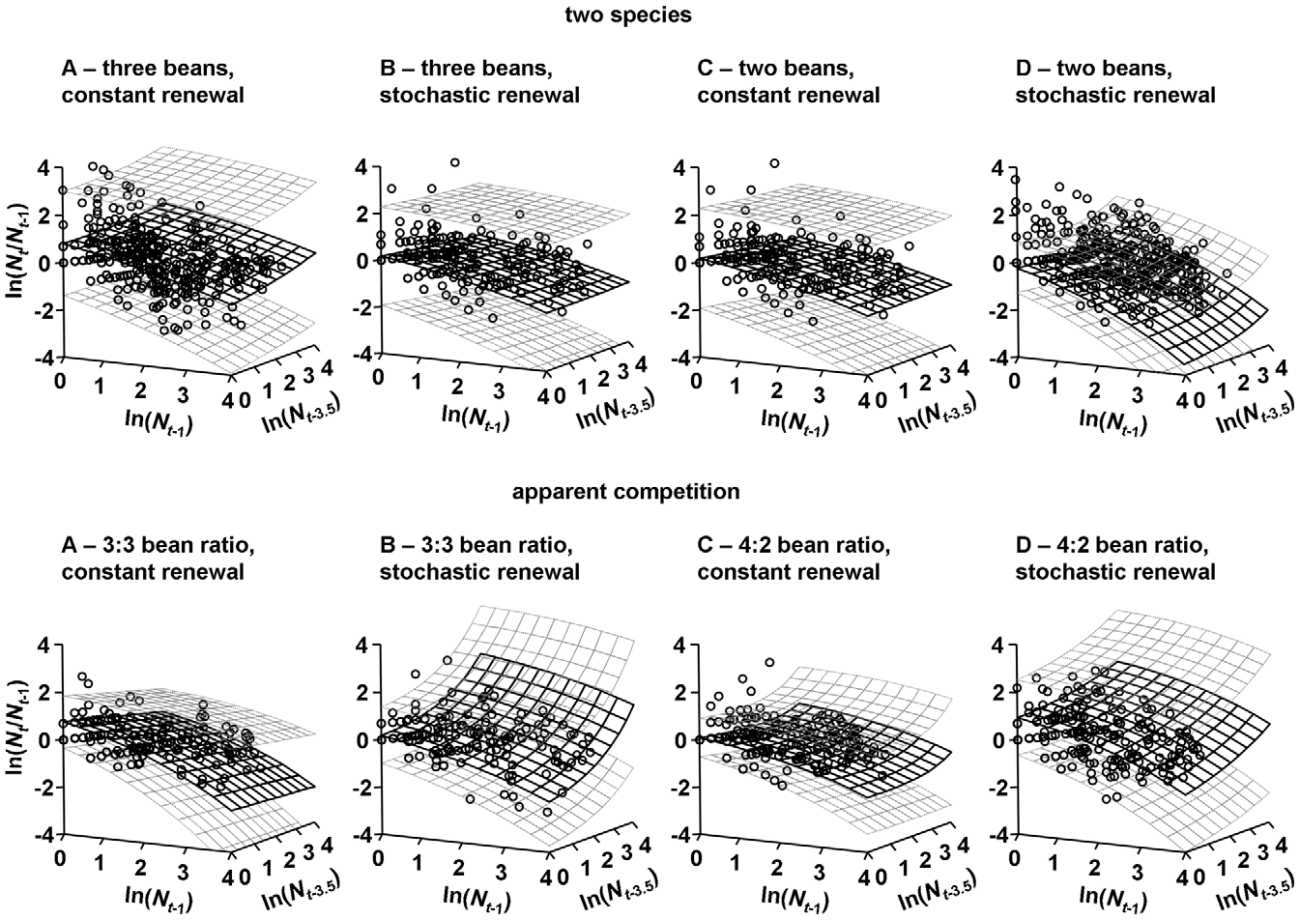
Predicted determinants of temporal density dependence in net reproductive rate, 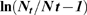, for *C. maculatus*. Using hierarchical mixed effects models, patchwise net reproductive rate was regressed against patchwise adult abundance covariates, 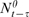, lagged by τ weeks, with their respective optimised Box-Cox transformations, *θ*. The two orthogonal lagged abundance covariates represent the best fitting within generation time lagged abundance (τ = 1 week) and one generation time lagged abundance, (τ = 3.5 weeks). Solid wireframe: maximum likelihood estimates. Grey, dotted wireframe: 95% confidence intervals.

Hierarchical spatiotemporal analysis successfully recovered the expected host regulatory dynamics in all cases. Typically, the host-parasitoid dynamics were of dimension (the number of limiting mechanisms with clearly distinguishable differences in time delay) two, regardless of resource renewal regime. That is, the patterns of abundance are components of previous abundances at two different time lags (Figure 3). Net reproductive rate was significantly negatively correlated with abundance over a one week time lag (density dependence operates). In addition, a second time lag of approximately one generation (3.5 weeks) also influences the metapopulation dynamics. Here, net reproductive rate is significantly positively density dependent. We suggest that this is indirect evidence of an Allee effect [23], [24]. This effect, likely the result of mate scarcity at very low population size, destabilises dynamics and increases extinction risks in otherwise already vulnerable, small populations.

### Spatial analysis

After accounting for density-dependent processes, the patchwise (negative) correlation between host abundances under each experimental treatment, are shown in table 1. The presence of an alternative host (two *vs*. three species assemblage) resulted in a significant increase in the degree of asynchrony in the local host population dynamics between patches (log odds ratio (SE) = 0.948 (0.318), *F*_1, 7_ = 14.2, *p* = 0.0093). Neither average resource level (*F*_1, 6_ = 0.622, *p* = 0.47), nor resource stochasticity (*F*_1, 4_ = 0.297, *p* = 0.62) were associated with asynchrony (negative correlation) in the population dynamics amongst patches. Although the superior apparent competitor persisted indefinitely, censuses were curtailed when the inferior host was driven to extinction. Therefore, we controlled for assemblage persistence, which also did not explain a significant amount of deviance in estimated spatial correlation (*F*_1, 5_ = 1.54, *p* = 0.28).

**Table 1:** Estimated patchwise spatial correlation, *ρ* 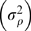, in host population dynamics.

	environmental treatment				
assemblage	Two species	a – three beans, constant renewal	b – three beans, stochastic renewal	c – two beans, constant renewal	d – two beans, stochastic renewal
		**−0.0294(0.0023)**	**−0.0127 (0.0124)**	**−0.0051 (0.0174)**	**−0.0399 (0.0024)**
	Apparent competition	a – 3∶3 bean ratio, constant renewal	b – 3∶3 bean ratio, stochastic renewal	c – 4∶2 bean ratio, constant renewal	d – 4∶2 bean ratio, stochastic renewal
		**−0.0650 (0.0021)**	**−0.0591 (0.0028)**	**−0.0572 (0.0025)**	**−0.0433 (0.0071)**

Patch level symmetric variance-covariance matrices were included in hierarchical (patches nested within replicate metapopulations) mixed effects models of density dependence in net reproductive rate, 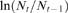. Negative synchrony was consistently greater under apparent competition (bottom row) than in the two species host-parasitoid metapopulations (top row). The effect of assemblage complexity outweighed the effects of resource renewal treatment (columns).

With an experimental design of patches nested within replicate metapopulations, we found that variation between average patch abundances (host: *σ_patch_* = 0.253, parasitoid: *σ_patch_* = 0.541) was quite substantial, compared to residual error (host: *σ_error_* = 0.437, parasitoid: *σ_error_* = 1.01), whereas between replicate variation was very low (host, parasitoid: *σ_replicate_*<0.001). This suggests a high degree of spatial heterogeneity within metapopulations, which we explore in the following section.

In order to ascertain the form of the spatial covariance evident in our experimental metapopulations, we produced separate empirical semivariograms [25] for each replicate metapopulation at each time point. Independent, weekly, semivariograms, 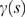, were constructed using standardised residuals of first-differenced time series of adult *C. maculatus* abundances from each patch. In four patch, square lattice metapopulations, *s* = 1 describes adjacent patches, *s* = 2 describes diametrically opposite patches (no diagonal dispersal was possible, and it was assumed occupants took the shortest possible route between patches). Paired Wilcoxon tests were applied to the resulting time series of asynchrony values, 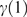 *vs*. 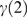, separately for each metapopulation. This supported the hypothesis that the degree of spatial correlation did not change with distance between patches: in 32 independent paired Wilcoxon tests (four resource renewal treatments × two species combinations × four replicate metapopulations) the mean p-value was 0.477, with only two p-values less than 0.05 (*p* = 0.033 and *p* = 0.045).

### Simulation model

**Figure 4:**
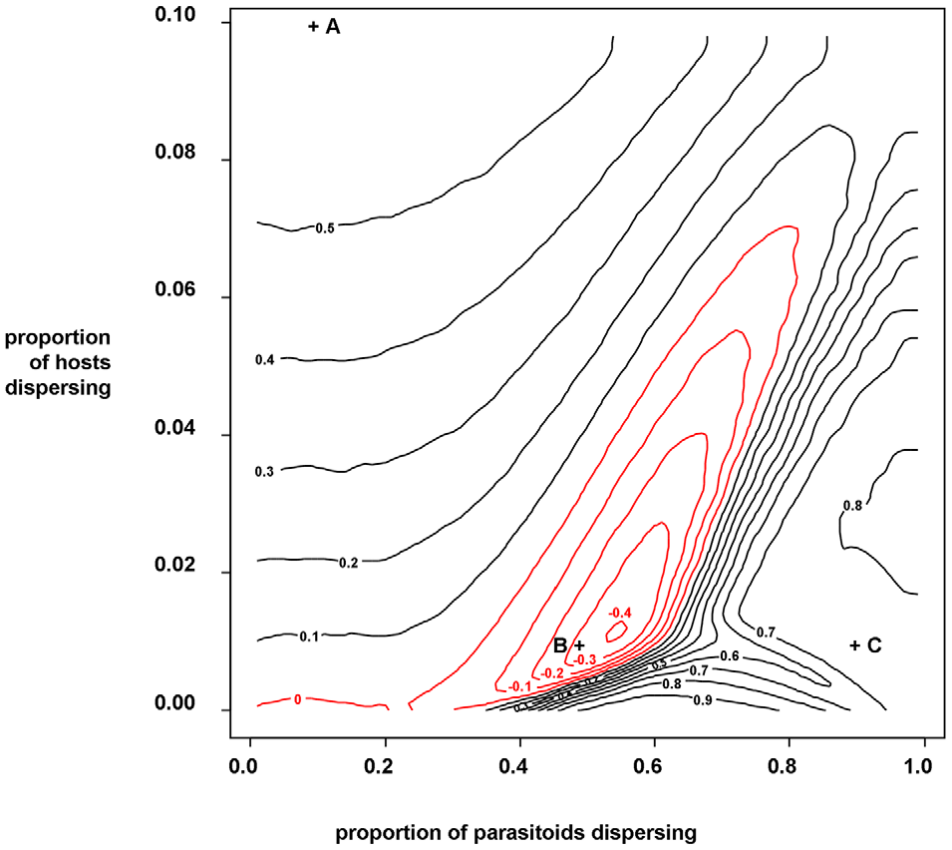
Predicted spatial correlations in local host abundances between habitat patches, under a range of host and parasitoid dispersal scenarios. The contours show common levels of correlation coefficient; black contours indicate positive correlation, red contours indicate negative correlation. Locations (+) labelled ‘a’ (10% host dispersal; 10% parasitoid dispersal), ‘b’ (1% host dispersal; 50% parasitoid dispersal) and ‘c’ (1% host dispersal; 90% parasitoid dispersal) relate to upper, middle and lower panels in figure 5 respectively.

**Figure 5:**
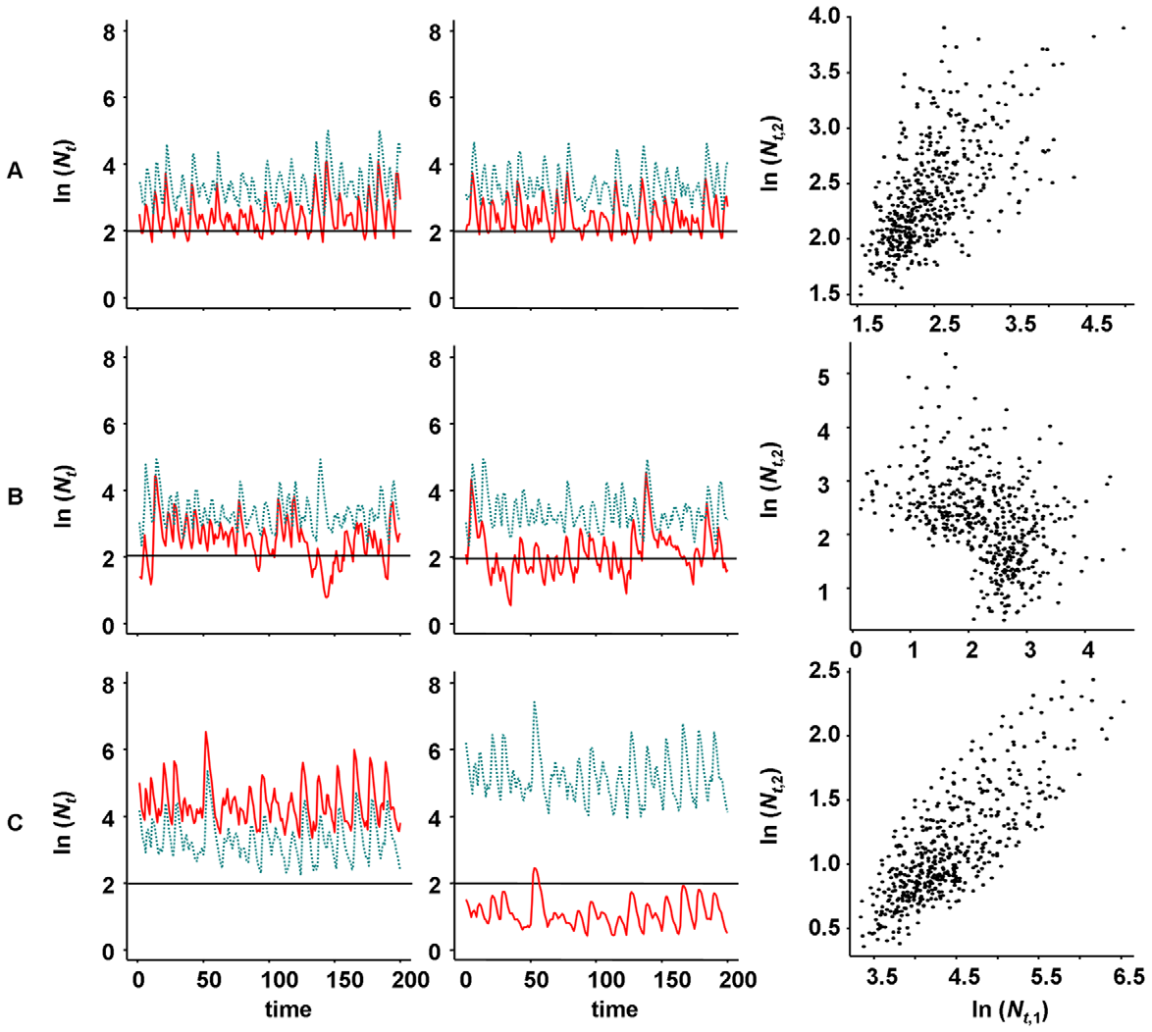
Representative time series of simulated host (solid, red line) and parasitoid (dotted, blue line abundances from two patch metapopulations (left and middle panels). Panels at right show host abundances in patch 1 (left panels) against patch 2 (middle panels). Row (a): 10% host dispersal; 10% parasitoid dispersal; row (b): 1% host dispersal; 50% parasitoid dispersal; and row (c): 1% host dispersal; 90% parasitoid dispersal.

Host-parasitoid dynamics were generated using a Nicholson-Bailey model (Equations 2 & 3), where demographic parameters were set to result in stable equilibrium dynamics so that populations only fluctuated due to stochastic perturbation. A range of proportions of hosts and parasitoids dispersing between the limiting case of two identical habitat patches were investigated to simulate noisy metapopulation dynamics. Figure 4 shows the range of positive and negative spatial correlation in host abundance that result from this system, with representative simulated time series shown in figure 5.

In order to explore the effects of parasitoid dispersal on the susceptibility of metapopulations to regional extinction, we investigated the frequency with which local host populations fell below an arbitrary abundance threshold, 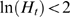. We allowed 1% of hosts to disperse at each time step and compared the effects of 10% (positively correlated local host abundances) *vs*. 50% (negatively correlated local host abundances) parasitoid dispersal. Parasitoid dispersal had no effect on mean local host abundance (*t*_198_ = 0.171, *p* = 0.86). Greater parasitoid dispersal significantly increased the number of times that one or other, but not both, local host populations were driven below the abundance threshold (log odds ratio (SE) = 1.81 (0.0712), *t*_198_ = 25.5, *p*<0.0001), by increasing temporal variance in local host abundance. However, the average length of time until both local host abundances were simultaneously driven below the threshold (regional extinction) was significantly longer with 50%, compared to 10%, parasitoid dispersal (log odds ratio (SE) = 1.21 (0.0549), *t*_198_ = 22.0, *p*<0.0001), due to asynchrony in local host populations, between habitat patches.

We compared these patch occupancy results with the equivalent simulation where host populations are limited by intraspecific competition (logistic density dependence), with no parasitoids present. The carrying capacity was set at the equilibrium size reached in the host-parasitoid simulations (by setting noise to zero). Positive correlation between local abundances was found for all proportions of hosts dispersing. In contrast to varying parasitoid dispersal, when we increased the proportion of hosts dispersing from 1% (low abundance correlation) to 10% (high abundance correlation), average population size was decreased (log odds ratio (SE) = −0.0110 (0.00107), *t*_198_ = 10.2, *p*<0.0001) but the number of times a single patch (but not both) was vacant also decreased (log odds ratio (SE) = −0.994 (0.0349), *t*_198_ = 28.5, *p*<0.0001), due to lower temporal variation. However, the result was a small but significant decrease in regional persistence time with higher host dispersal (log odds ratio (SE) = −0.160 (0.0322), *t*_198_ = 4.96, *p*<0.0001).

## Discussion

The key experimental result here is that apparent competition, mediated by a shared natural enemy, was associated with an increase in negative spatial synchrony, when compared to the two species, host-parasitoid interaction. This effect of assemblage complexity overshadowed a range of additional environmental covariates such as resource availability and supply. Such asynchrony between local population fluctuations is well known to facilitate recolonization of locally extinct habitat patches, as well as maintain regional metapopulation persistence [26]–[28].

In our microcosm system, the presence of an alternative, but not directly interacting, host provides a reservoir for parasitoids, allowing a sustained increased level of foraging on the superior host competitor [15], [16]. In the absence of spatial structure, it is well established that one of the host species is rapidly driven extinct [15], [16]. However, here we show that spatial processes, modified by assemblage complexity and the presence of a shared natural enemy, can potentially provide a mechanism protecting metapopulations from this regional extinction. By comparing between two species, host-parasitoid and three species, apparent competitive assemblages, we found that increased parasitism did not result in a greater chance of local host population extinction through stochastic fluctuations. We conclude that since assemblage complexity does not influence local extinction risk, the resulting regional asynchrony can provide a protective mechanism against extinction.

To explore how assemblage complexity and metapopulation structure can interact to affect species persistence, we analysed a range of environmental microcosm treatments. As expected, environmental stochasticity increased variation in host abundance. However, we found that noise at the lowest trophic level (host resource) does not filter up through the host-parasitoid interaction. It is well understood that single species populations can modify, or filter, the effects of environmental noise to result in observed species abundances [29], [30]. However, how noise is filtered through trophic interactions to determine predator distribution is less well resolved, particularly in spatially subdivided environments. Here, by foraging across several patches, the highly dispersive parasitoids average out noise to a greater extent than the less mobile hosts. Whereas, higher average host resource level did not influence levels of variation in the host population but, unexpectedly, did increase parasitoid fluctuations: we suggest that greater host availability may have destabilised parasitoid dynamics through the ‘paradox of enrichment’ – rather than conferring greater population stability, elevated resource levels may promote high order limit cycles and chaotic dynamics by increasing net reproductive rate [31], [32].

Given that environmental noise was independent across habitat patches, spatial processes were mediated by adult host and/or parasitoid dispersal. If host dispersal had been principally responsible for the observed relationship between local population dynamics, we would have expected patch dynamics to be broadly in phase, and so positively correlated [9], [33]. Whereas, if synchrony in the host population dynamics was driven by parasitoids, the time-delayed response resulting from this trophic interaction would cause local population dynamics to shift out of phase. This mechanism corresponds to our empirical result of negative spatial correlation amongst patches and would account for the greater degree of asynchrony under apparent competition, due to the associated increase in parasitoid attack. Our results are seemingly at odds with recent findings using bacterial-phage interactions [34], in which the presence of a parasite (phage) was associated with stronger positive spatial synchrony between host bacteria populations. It is well understood that natural enemies can generate [35] and phase lock [36] deterministic population cycles. However, previously we have shown that local host dynamics do not involve pronounced limit cycles in this system [14]. Here, spatial modification of stochastic processes by freely roaming parasitoids accounts for the observed spatial asynchrony.

We explored the generality of this pattern of predator-driven prey asynchrony in a resource-consumer metapopulation model and demonstrate how parasitism can affect extinction risk in a patchy habitat. As expected, host dispersal synchronised host abundance between patches. However, increasing parasitoid dispersal disrupts the synchronising effects of host dispersal, driving negative correlation between host populations. This asynchrony occurs through the lag in host regulation introduced by the host-parasitoid trophic interaction. With increasing host dispersal, a higher level of parasitoid dispersal was necessary to drive negative correlation between host populations. However, very high levels of parasitoid dispersal destabilised metapopulation dynamics, resulting in suppression of hosts in one (random) habitat patch, establishing source-sink dynamics. In this case, host abundance in the sink patch was maintained by host immigration, so was positively correlated with source patch host abundance. These findings corroborate our experimental results and demonstrate that natural enemy interactions can produce asynchronous, as well as synchronous spatial patterns of host abundance.

Our results have implications for conservation of small populations in fragmented habitats. Recolonization events of extinct patches are enhanced by asynchrony between local dynamics, and we have quantified how asynchrony is determined by the complexity of the community and the impact of consumers. Paradoxically, by enhancing and maintaining asynchronous local population dynamics between habitat patches, predation may provide a mechanism protecting small metapopulations from stochastic extinction events. Since the degree of asynchrony in our empirical, resource-consumer metapopulations is dependent on trophic interactions and assemblage complexity, it certainly follows that reducing community complexity, in particular in small, fragmented habitats, will reduce the efficacy of metapopulation rescue effects. In a stochastic world, small metapopulations are likely to be even more vulnerable to extinction than previously thought, as reductions in biodiversity and ecosystem function, together with habitat degradation and fragmentation, affect species persistence.
